# Angular surface plasmon resonance-based sensor with a silver nanocomposite layer for effective water pollution detection

**DOI:** 10.1038/s41598-023-48837-4

**Published:** 2023-12-08

**Authors:** Fatma A. Sayed, Hussein A. Elsayed, M. Al-Dossari, M. F. Eissa, Ahmed Mehaney, Arafa H. Aly

**Affiliations:** 1 TH-PPM Group, Physics Department, Faculty of Sciences, Beni-Suef University, Beni Suef, 62514 Egypt; 2 Department of Physics, Faculty of Science, King Khalid University, 62529 Abha, Saudi Arabia

**Keywords:** Materials science, Optics and photonics, Physics

## Abstract

For sensing various samples of polluted water and various sodium chloride concentrations using an angular surface plasmon resonance (ASPR), we have introduced a conventional structure and a hybrid heterostructure in the current research. The suggested structures are composed of silver metal, dielectric layers, silver nanocomposite, and a sensing medium. The reflectance spectra of all structures in the visible region were obtained through the utilization of the transfer matrix method by using the angular interrogation method depending on the Kretschmann configuration. Through our findings, five substrate parameters have been optimized to attain the utmost level of sensitivity across all structures: the thickness of Ag-metal, the type and thickness of dielectric materials, the host material type and the volume fraction of nanoparticles for the nanocomposite layer. In this regard, the suggested sensor provides excellent performance with a sensitivity of 448.1°$/\text{RIU}$, signal-to-noise ratio of 0.787, sensor resolution of 0.284°, and figure of merit of 78.766 RIU^−1^. Therefore, we believe that the introduced design of our ASPR sensor presents a good candidate for an accurate and efficient detection of low concentrations of contaminated water and sodium chloride as well.

## Introduction

Ritchie successfully observed surface plasmon resonance (SPR) theoretically in 1953^1^, after the first attempt in 1902^2^. The phenomenon of SPR is defined as the stimulation of the SP-wave by the evanescent field of incident p-polarized light that is exponentially decaying along the interface of the metal dielectric^3^. The resonance requirement is satisfied when the frequency of the incident light coincides with the frequency of the SP wave. These waves may serve as an indicator to identify an analyte's changes in the refractive index (RI) based on their interaction with the surface of the detecting medium^3^. This phenomenon has been extensively used in a wide range of applications, including sensor technology, nonlinear optics, spectroscopy, optical modulators, and microscopy. Along with additional benefits including reliable and real-time detection capabilities, the SPR-based biosensors have a label-free platform that enables flexible and extremely sensitive detection^4^.

Meanwhile, the attenuated total reflection (ATR) coupler method is commonly used for exciting SP waves in SPR sensors. This method utilizes two configurations: the Otto and Kretschmann types. The Otto type involves the immersion of the sensing medium between a prism and a metallic layer, while in the Kretschmann type, the sensing medium is placed in direct contact with a thin metallic layer that is directly deposited on the base of a prism with a high dielectric constant^3^. Furthermore, the Kretschmann arrangement also affords an improved value of signal to noise ratio, thereby permitting the SP waves to permeate the sensing medium, resulting in heightened interactions with the analyte^5^. Therefore, the Kretschmann configuration became more practical as it does not also require control of the sensing medium gap^3,6^.

In the last 10 years, SPR-based biosensors have received a lot of interest due to their heightened sensitivity. Dai et al. suggested a SPR sensor made of SnSe and Ag with a sensitivity of 176°/$\text{RIU}$, and a low reflectance of 0.4674^7^. H. A. Zain et al. proposed an SPR sensor using a Kretschmann setup with a gold-coated prism for food safety^8^. K. A. Meradi et al. tried to modify the conventional SPR sensor by adding a 1D-photonic bandgap array instead of the ordinary metallic film in SP sensors^9^. However, these traditional structures have a low sensitivity, big full width at half maximum (FWHM), and low-quality factor.

Recent research has revealed that hybrid heterostructures are becoming increasingly popular in contemporary times for the implementation of SPR biosensors due to their exceptional sensing capabilities. Hybrid heterostructures are structures that are formed by combining different materials or layers, resulting in a novel structure that possesses unique properties^6^. Md. Shamim Anowe et al. conducted a study to explore the sensitivity of SPR biosensors using hybrid heterostructures^5^. The research has revealed that the SPR biosensor sensitivity with hybrid heterostructures is significantly higher, at least 1.67 times, compared with the conventional non-hybrid structures^6^. Shuwen Zeng et al. suggested an SPR sensor utilizing a hybrid structure of graphene and MoS_2_, which has demonstrated exceptional sensitivity in detecting molecules. The suggested system exhibits a phase-sensitivity augmentation factor of over 500-fold when compared with the SPR sensing approach without the graphene-MoS2 coating or with only graphene coating^10^. Shuaiwen Gan et al. posited the utilization of 2D franckeite nanosheets for the development of a SPR sensor to improve its sensitivity. The introduced sensor exhibits a 62% improvement in sensitivity as compared with conventional SPR biosensors^11^. The studies mentioned above have contributed valuable insights towards the development of a hybrid SPR biosensor exhibiting heightened sensitivity.

As a result, we describe in this paper a novel design of enhanced SPR biosensors based on hybrid heterostructures, which combines conventional SPR biosensors with silver nanocomposite and other dielectric materials. These hybrid heterostructures have been found to have improved sensing properties in comparison with their non-hybrid counterparts, making them attractive candidates for use in applications for detecting environmental contamination. In this regard, the numerical findings introduced the ability of improving the performance of the designed SPR sensor in the vicinity of optimizing the governing properties of the considered materials. Notably, the optimiziation of the permittvity of the hosting material and volume fraction of Ag’s nanoparticles for the nanocomposite layer could increase the sensitivity of the designed sensor up to 488°/RIU. The inclusion of a nancoposite layer could exhibit a 54% improvement compared to the conventional SPR biosensors. Meanwhile, we examined the ability of our sensor to detect some different water pollutants such as water from a dirty pond, water that has been chemically tainted, drainage water, and water that contains sodium chloride in varying amounts.

## The optical properties of the materials of the hybrid heterostructures

The hybrid SPR heterostructures as well as some additional traditional angular SPR-based biosensing designs were presented in this study. The traditional SPR sensor is based on the metal layer which is utilized to produce surface plasmon. The low absorbability of metal is the reason behind the poor sensitivity of the traditional SPR sensor. Therefore, numerous articles have presented innovative materials that have the potential to substantially enhance the sensitivity of SPR biosensors^6,12–15^.

**Figure 1:**
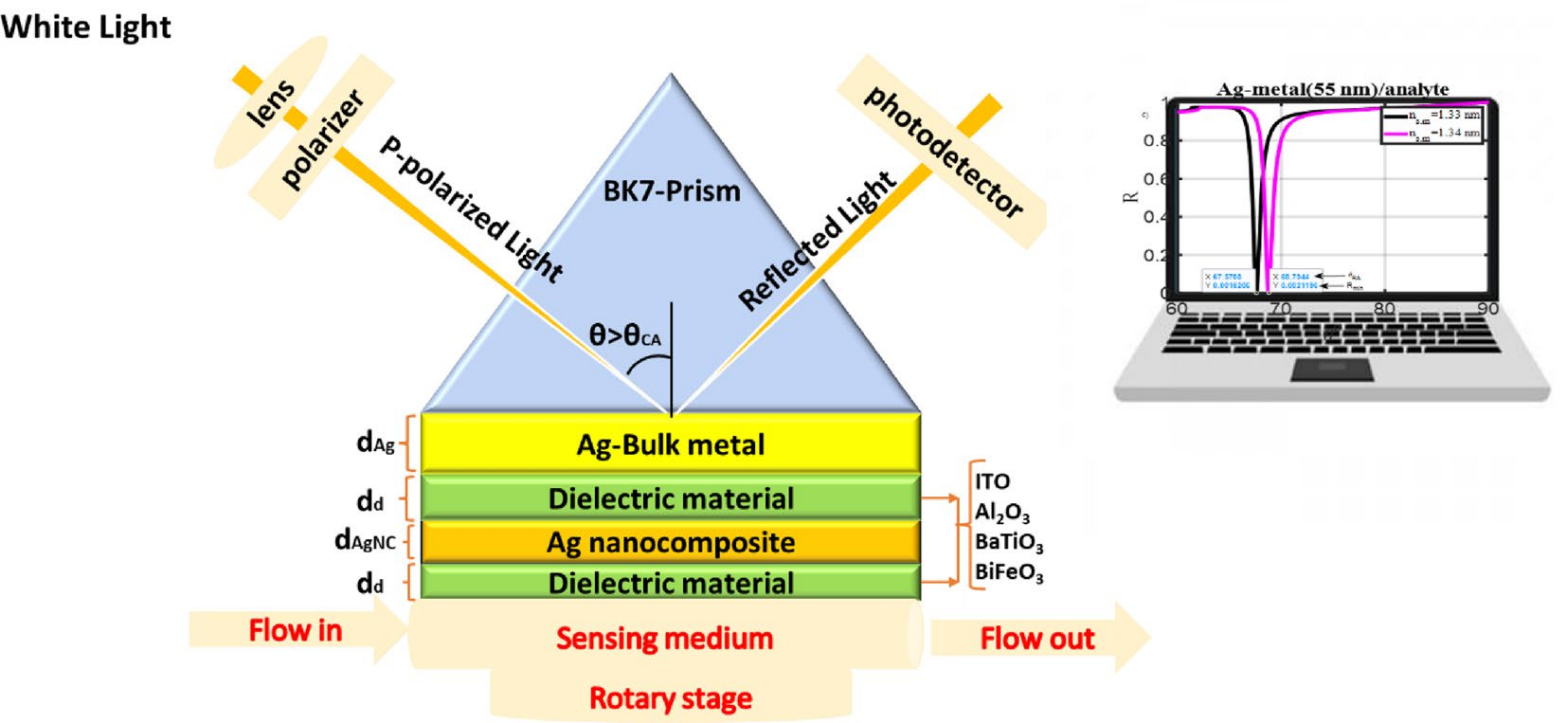
Schematic diagram of the proposed angular hybrid heterostructure SPR sensor.

The diagram in Fig. 1 illustrates the suggested ASPR sensor which incorporates the use of a dielectric material, specifically BiFeO_3_. This sensor is comprised of five layers, starting with a BK7 dielectric prism which is utilized to match the resonance between the incident wave and SP wave. Depending on the wavelength of the incident light, the BK7 dielectric prism's refractive index changes^16^. Following the prism is a thin layer of metal made of Ag that supports the SP wave at the metal-dielectric wave. According to the Drude model, this metal layer's dielectric constant likewise fluctuates with wavelength^17^. A dielectric layer is used to cover the metal layer to enhance the SPR sensor's performance. The performance of the SPR sensor is improved by covering the metal layer with a dielectric layer. Then, this layer is in contact with the sensing medium, which has a refractive index of between 1.33 and 1.34 RIU.

Here, we will show that the angular SPR sensor can exhibit high sensitivity when utilizing hybrid heterostructures consisting of silver-metal, thin film dielectric material BiFeO_3_, and silver nanocomposite. Table 1 contains a list of the details about the structures for each of the design layers, from structure-1 to structure-11.

**Table 1:** Structures of several conventional and hybrid heterostructures SPR multilayer sensors.

Structure	Details of SPR sensor
Structure 1	Prism/Ag/sensing medium (S.m)
Structure 2	Prism/Ag/ITO/S.m
Structure 3	Prism/Ag/Al_2_O_3_/S.m
Structure 4	Prism/Ag/BaTiO_3_/S.m
Structure 5	Prism/Ag/BiFeO_3_/S.m
Structure 6	Prism/Ag/BiFeO_3_/Ag/S.m
Structure 7	Prism/Ag/BiFeO_3_/AgNC/ITO/ S.m
Structure 8	Prism/Ag/BiFeO_3_/AgNC/Al_2_O_3_/ S.m
Structure 9	Prism/Ag/ BiFeO_3_/AgNC/BaTiO_3_/ S.m
Structure 10	Prism/Ag/BiFeO_3_/AgNC/BiFeO_3_/ S.m
Structure 11	Prism/Ag/BiFeO_3_/AgNC/BiFeO_3_/AgNC/BiFeO_3_/S.m

The optical characteristics of each layer in the suggested SPR sensor are then provided.

### BK7-prism

It is important to remember that the prism's refractive index has a big influence on how the sensor is made because it affects the evanescent wave's strength and angle of incidence. Following are the formulas for the BK7-prism's refractive index (RI)^18^:

1 $${{{\text{n}}}}_{{{\text{P}}}}=\frac{1.03961212{\lambda }^{2}}{{\lambda }^{2}-0.00600069867 }+\frac{0.231792344{\lambda }^{2}}{{\lambda }^{2}-0.0200179144}+\frac{1.01046945{\lambda }^{2}}{{\lambda }^{2}-103.560653}+1$$

In this case, the proposed SPR sensor's operating wavelength is 633 nm.

### Ag-metal

Ag metal, also known as silver metal, is a widely utilized plasmonic metal in SPR-based sensors. It possesses sharper and more intense SPR bands in comparison to gold (Au) and exhibits low optical loss in visible and near-infrared spectral bands, rendering it an ideal material for plasmonics. The Ag-metal's refractive index can be calculated according to the model as follows^14,19^:

2 $${{\text{n}}}_{{\text{Ag}}}=\sqrt{1-\frac{{\uplambda }^{2}{\uplambda }_{{\text{c}}}}{{\uplambda }_{{\text{p}}}^{2}({\uplambda }_{{\text{c}}}+\mathrm{\lambda i})}}$$

Silver's wavelengths with respect to plasma and collision frequencies are defined by the values of ${\uplambda }_{{\text{p}}}$ = 8.9342 × 10^–6^ m, and ${\uplambda }_{{\text{c}}}$ = 1.6826 × 10^–7^ m, respectively,^18,20^.

### Dielectric materials

Ag-metal may be subjected to oxidation issues and significant losses due to its surface roughness^18,19^. A protective layer could be used over the Ag layer to have excellent performance. This protecting layer acts as a barrier against oxidation and corrosion-related issues that could compromise the SPR sensor's performance^4^. Proper selection of the protecting layer is critical in ensuring that the SPR sensor's performance is not compromised. Therefore, a comparison is introduced between the different types of dielectric materials, and the material that gives the excellent performance of our sensor was selected. Table 2 shows the refractive indices of the various dielectric layers.

**Table 2:** The refractive index of the material used at wavelength 633 nm.

Material used	Refractive index (RI)	Ref
BK7-prism	1.151	^28^
Ag	0.05626 + (4.2776i)	^29^
BaTiO_3_	2.4042	^30^
BiFeO_3_	2.9680	^31^
Al_2_O_3_	1.77	^32^
ITO	1.858 + (0.058i)	^33^
Sensing medium	1.33 to 1.34	

### Silver nanocomposite

A silver nanocomposite refers to a composite material that comprises silver nanoparticles within a matrix consisting of another dielectric material. The characteristics of the nanocomposite are influenced by several factors, including the size, and the filling fraction of the silver nanoparticles, as well as the properties of the matrix (host) material^21^. These characteristics lead to some properties different compared to bulk metals.

To overcome the limitations that are imposed on dispersive materials (such as metals), scientists have recently resorted to nanocomposite materials. Materials with nanocomposite structures may exhibit peculiar optical properties in particular. It is possible to create composite materials by dispersing nanoparticles within a host material. For the creation and design of many optical systems, composite materials containing metallic nanoparticles are quite desired. Nanocomposite materials may therefore be of considerable interest in the field of ASPR as a result of their function in the tunable of the sensitivity to the polarization mode. The use of nanocomposite materials in optical and biological sensing applications has also gained considerable interest^22^.

The nanocomposite layer's frequency-dependent refractive index can be explained by the Maxwell–Garnett model. Here, we assume that the nanocomposite material is formed of silver nanoparticles that are randomly dispersed inside a matrix of a transparent dielectric material. This model specifies the effective permittivity (ε_eff_) of the nanocomposite layer as the following equation^22^:

3 $${\upvarepsilon }_{{\text{eff}}}=\frac{{2\upvarepsilon }_{{\text{d}}}{\text{V}}\left({\upvarepsilon }_{{\text{Ag}}}-{\upvarepsilon }_{{\text{d}}}\right)+{\upvarepsilon }_{{\text{d}}}\left({\upvarepsilon }_{{\text{Ag}}}+2{\upvarepsilon }_{{\text{d}}}\right)}{{2\upvarepsilon }_{{\text{d}}}+{\upvarepsilon }_{{\text{Ag}}}+{\text{V}}\left({\upvarepsilon }_{{\text{d}}}-{\upvarepsilon }_{{\text{Ag}}}\right)}$$

where ${\text{V}}$ denotes the volume fraction of the nanoparticles and ${\upvarepsilon }_{{\text{d}}}$ represents the permittivity of the host dielectric material. ${\upvarepsilon }_{{\text{Ag}}}={{{\text{n}}}_{{\text{Ag}}}}^{2}$ represents the permittivity of the silver nanoparticles. The refractive index of the silver nanocomposite:

4 $${{\text{n}}}_{{\text{AgNC}}}=\sqrt{{\upvarepsilon }_{{\text{eff}}}}$$

## The proposed sensor's feasibility in experiments

**Figure 2:**
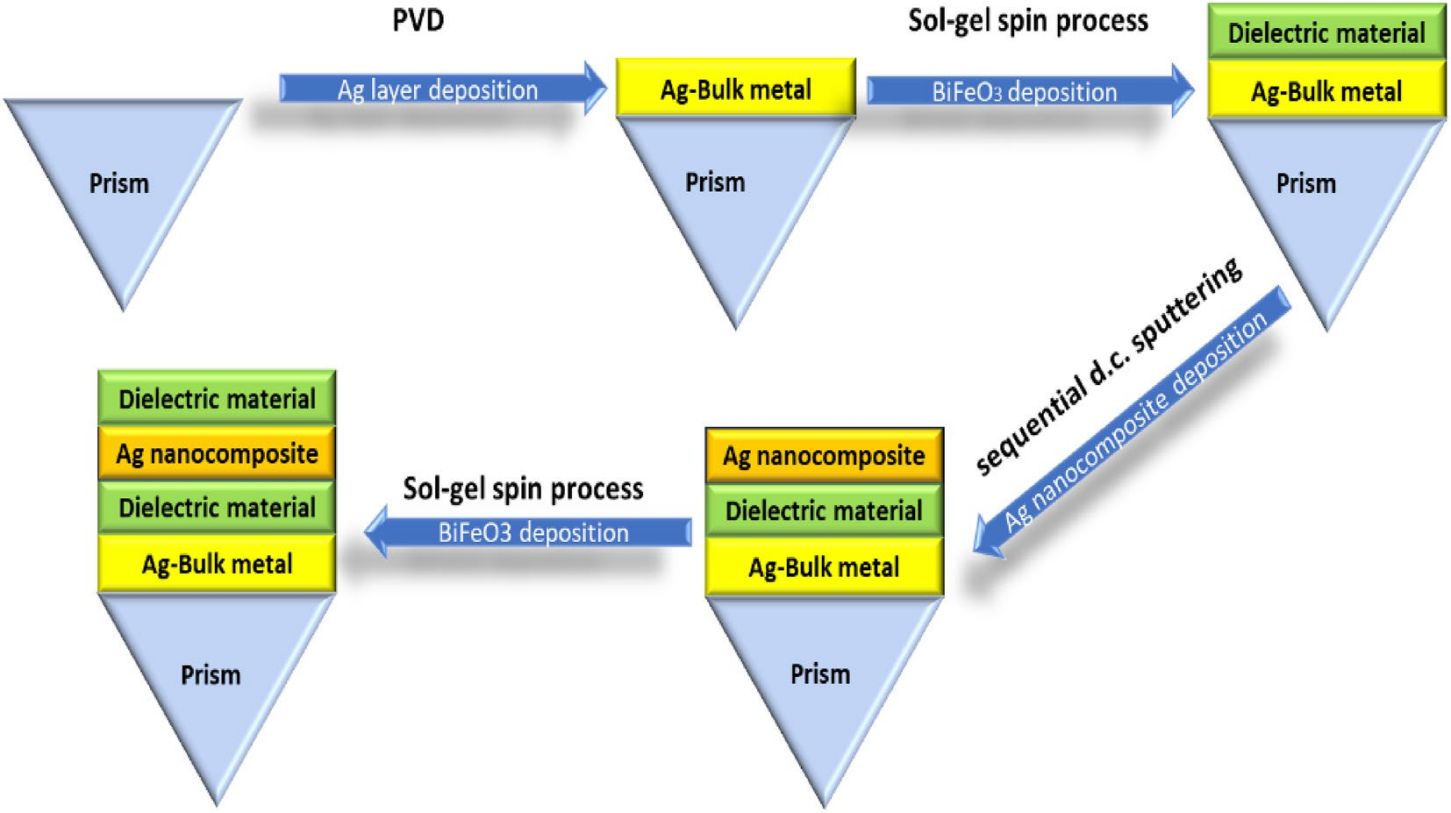
Possible fabrication processes for the suggested design.

Multilayer conventional and hybrid heterostructures can be conveniently synthesized on the prism surface through the implementation of the subsequent procedures: BK7-prism serves as the substrate for the sensor. The first step in the fabrication process is cleaning the prism's surface up to 4–5 times with methanol, acetone vapors, and deionized water. Then, the physical vapor deposition (PVD) method is utilized to deposit the layer of Ag metal on its base. The Ag layer thickness is influenced by the particle sputtering deposition time. The Ag layer thickness in the sol–gel spin method is thus dependent on the duration of the particle sputtering deposition. The BiFeO_3_ layer would next be applied using the sol-gel spin method, followed by the sequential d.c. sputtering method or high-pressure sputtering technique to deposit Ag nanocomposite layer^21^. Finally, the sol-gel spin method is used to deposit the dielectric material layer. Figure 2 illustrates the range of fabrication techniques that could be used to create the recommended design.

There are three fundamental steps in the sensing process: the sample cell should first be firmly fastened to the detecting structure's surface. Next, slowly add the analyte into the sample cell using a pump. The flow of the analyte can then be detected using a resonance signal. Additionally, a device made up of a lens, polarizer, and collimator can be used to focus the monochromatic light from the He-Ne laser toward the prism^10^. An instrument called a photodetector is then utilized to measure the intensity of the light that has been reflected.

## The proposed SPR-sensor's basic principle

Based on the Kretschmann type, the method of ATR is employed for exciting resonance in the SP wave within the SPR sensors in which, the placement of a thin metallic layer possessing a dielectric constant, denoted as ε_m_, directly on the base of a high dielectric constant prism, denoted as ε_0_. After that, the medium used for sensing (s.m) is brought into touch with the metallic layer, allowing the total internal reflection's evanescent wave to pass through it and excite the SP wave at the surface plasmon wave at the metal/sensing medium interface^3^.

The resonance condition given by Eq. (5) is satisfied when the P-polarized incident light's wave vector coincides with the SP wave propagating along the metal-sensing medium interface.

5 $${{\text{K}}}_{{\text{in}}}={{\text{K}}}_{{\text{SPW}}}$$

6 $$\frac{2\uppi }{\uplambda }{{\text{n}}}_{{\text{P}}}{\text{sin}}({\uptheta }_{{\text{in}}})=\frac{2\uppi }{\uplambda }\sqrt{{{{\text{n}}}_{{\text{m}}}^{2}{{\text{n}}}_{{\text{d}}}^{2}}/ {{{\text{n}}}_{{\text{m}}}^{2}+{{\text{n}}}_{{\text{d}}}^{2}}}$$

where, ${{\text{K}}}_{{\text{in}}}, {{\text{K}}}_{{\text{SPW}}},\uplambda , {{\text{n}}}_{{\text{P}}}, {\uptheta }_{{\text{in}}},{{\text{n}}}_{{\text{m}}},\mathrm{ and }{{\text{n}}}_{{\text{d}}}$ are the P-polarized light's incident wave vector, the wave vector of the SP wave, the incident P-polarized light's wavelength, the prism's refractive index, the laser beam's incidence angle at the prism-metal interface, the silver metal's refractive index, and the sensing medium's refractive index, respectively.

The resonance condition is determined by each of these parameters. The incident angle is referred to as the resonance angle under this condition and is given by the following Eq. (7).

7 $${\uptheta }_{{\text{SPR}}}={{\text{sin}}}^{-1}(\frac{1}{{{\text{n}}}_{{\text{P}}}}\sqrt{{{{\text{n}}}_{{\text{m}}}^{2}{{\text{n}}}_{{\text{d}}}^{2}}/ {{{\text{n}}}_{{\text{m}}}^{2}+{{\text{n}}}_{{\text{d}}}^{2}}} )$$

P-polarized light's incidence on the prism-metal interface and its subsequent reflection according to the total internal reflection theory is used to measure the reflectivity of the light through an optical detector. The wavelength interrogation mode as a function of the incident light's wavelength with a fixed incident angle or the phase interrogation mode as a function of the incident angle are both used to measure the reflected light. A sharp dip is seen at the resonance angle or resonance wavelength in both modes. The sample's refractive index can be determined via the resonance angle^3,23^.

To establish resonance conditions, the employment of a laser source is deemed necessary. For this particular setup, it is proposed to utilize a He–Ne laser that has a wavelength of 633 nm. It is noteworthy that even a slight modification within the sensing medium's dielectric constant can result in a significant shift in the SPR curve. As a result, the resonance angle is significantly changed, which improves the SPR sensor's performance.

## Mathematical modeling for reflectance calculation

There are three different ways to derive an equation that describes the radiative characteristics of a multilayer, specifically in the Kretschmann configuration, such as reflectance and transmittance. These techniques include the transfer matrix method (TMM), the resultant wave method, and the field tracing approach. Of these techniques, the TMM is deemed as the most precise, since it entails no approximations^20,24^.

To calculate the transfer matrix for a given layer, it is necessary to determine both the phase shift and admittance. This can be achieved by following the subsequent steps:

8 $${{\text{S}}}_{{\text{j}}}=\frac{2\uppi }{\uplambda }{{\text{d}}}_{{\text{j}}}\sqrt{{{{\text{n}}}_{{\text{j}}}^{2}-({{\text{n}}}_{{\text{P}}}{\text{sin}}\left({\uptheta }_{{\text{in}}}\right))}^{2}}$$

9 $${{\text{P}}}_{{\text{j}}}=\frac{\sqrt{{{{\text{n}}}_{{\text{j}}}^{2}-({{\text{n}}}_{{\text{P}}}{\text{sin}}\left({\uptheta }_{{\text{in}}}\right))}^{2}}}{{{\text{n}}}_{{\text{j}}}^{2}}$$

where: ${{\text{S}}}_{{\text{j}}}$ represents the admittance, ${{\text{P}}}_{{\text{j}}}$ indicates the phase shift, ${{\text{n}}}_{{\text{j}}}$ the refractive index, and ${{\text{d}}}_{{\text{j}}}$ the thickness of layer j, respectively.

Figure 1 depicts the configuration of a SPR sensor consisting of four layers. At each interface of the layers, multiple reflections occur in response to incident light at the interface between the first layer of a prism. To calculate accurately the overall reflection/transmission computations, the accumulation of these reflections must be taken into account. The TMM, as depicted in Eq. (10), describes how a wave moves across medium j and towards medium (j + 1)^3^.

10 $${{\text{M}}}_{{\text{i}}}=\left(\begin{array}{cc}{{\text{cosS}}}_{{\text{i}}}& -\frac{{\text{i}}}{{{\text{P}}}_{{\text{i}}}}{{\text{sinS}}}_{{\text{i}}}\\ -{{\text{iP}}}_{{\text{i}}}{{\text{sinP}}}_{{\text{i}}}& {{\text{cosS}}}_{{\text{i}}}\end{array}\right)$$

The overall multilayer transfer matrix is obtained as a function of the transfer matrix ${{\text{M}}}_{{\text{i}}}$ for each layer, as shown below:

11 $${{\text{M}}}_{{\text{total}}}={\prod }_{{\text{j}}=2}^{{\text{m}}-1}\left(\begin{array}{cc}{{\text{m}}}_{11}& {{\text{m}}}_{12}\\ {{\text{m}}}_{21}& {{\text{m}}}_{22}\end{array}\right)$$

For a multilayer construction with m layers, the overall reflection R-coefficient is calculated as follows:

12 $${\text{r}}=\frac{\left({{\text{m}}}_{11}+{{\text{m}}}_{12}{{\text{P}}}_{{\text{s}}}\right){{\text{P}}}_{0}-\left({{\text{m}}}_{21}+{{\text{m}}}_{22}{{\text{P}}}_{{\text{s}}}\right)}{\left({{\text{m}}}_{11}+{{\text{m}}}_{12}{{\text{P}}}_{{\text{s}}}\right){{\text{P}}}_{0}+\left({{\text{m}}}_{21}+{{\text{m}}}_{22}{{\text{P}}}_{{\text{s}}}\right)}$$

wherein the context of the structure of length L, ${{\text{P}}}_{0}$ and ${{\text{P}}}_{{\text{s}}}$ are respectively denoted as the incident (z < 0) and exit (z > L) media. As seen in Fig. 1, the entirety of the thin film layers of the structures that have been taken into consideration for this study have been systematically arranged along the z-axis. The reflectivity of the SPR structure is then determined by:

13 $${\text{R}}={\left|{\text{r}}\right|}^{2}$$

## Parameters of the suggested sensor's performance

The sensitivity (S), quality factor (QF), full width at half maximum (FWHM), figure of merit (FoM), detection limit (DL), signal-to-noise ratio (SNR), and sensor resolution (SR) are the performance parameters of an SPR sensor that determine how well it performs. The suggested sensor would perform optimally if both S and QF are maximized while keeping the FWHM value to be a minimum value^18^.

### Sensitivity (S)

One of the factors that most significantly affects SPR sensor performance is sensitivity, which defines the sensor's capacity to identify the kind and concentration of the sample.

The definition of sensitivity for the proposed design is the change in the angular location of the reflectance dip (Δθ) or the change in the resonance wavelength of the reflectance dip ${\Delta \lambda }$ for the change in the refractive index of the sensing medium (Δn). It is mathematically defined as^4^:

14 $${\text{S}}=\frac{{\Delta \theta }}{{\Delta n}}\left({\circ}\text{/RIU}\right) ({\text{In-phase interrogation}}),\, ({\text{or}})$$

$${\text{S}}=\frac{{\Delta \lambda }}{{\Delta n}}\left({\text{nm}}\text{/RIU}\right) (\mathrm{In \, wavelength \, interrogation})$$

High sensitivity requires a significant change in the resonance angle (in phase interrogation) or resonance wavelength (in wavelength interrogation) with just a slight change in the sample's refractive index, thickness, and concentration^3^. In the following study, the performance parameters determined depend on the phase interrogation.

### Full width at half maximum (FWHM), and signal to noise ratio (SNR)

FWHM is a parameter that characterizes the width and sharpness of the reflectance curve. The FWHM is a measure of the difference observed between the resonance angle and reflectance at 50%. In the context of designing biosensors, it is desirable to have a relatively small FWHM to attain the best performance^25^. To conduct a thorough assessment of the SPR signal's efficacy, it is imperative to establish the SNR through appropriate mean^26^.

15 $${\text{SNR}}=\left(\frac{\Delta {\uptheta }_{{\text{SPR}}}}{{\text{FWHM}}}\right)$$

### Quality factor (QF)

The SPR sensor's quality factor is contingent upon the incidence angle wherein reflectance reaches its minimum level (R_min_). This angle is referred to as the resonance angle or the SPR angle (θ_SPR_). The aforementioned factor is characterized as follows.

16 $${\text{QF}}=\frac{{\uptheta }_{{\text{SPR}}}}{{\text{FWHM}}}$$

### Figure of merit (FoM)

FoM refers to the determination of the SPR sensor's resolution. A good sensor will have a high FOM value, which denotes a sharp FWHM spectrum and high sensitivity^26^.

17 $${\text{FoM}}=\frac{{\text{S}}}{{\text{FWHM}}}$$

### The detection limit (DL), and sensor resolution (SR)

18 $${\text{DL}}=\left(\frac{{\uptheta }_{{\text{SPR}}}}{20\mathrm{ S }({\text{QF}})}\right)$$

19 $${\text{SR}}=\left({\text{DL}}\right)\left({\text{S}}\right)$$

## Numerical results

The current study employs the transfer matrix approach to attain the reflectivity of a variety of conventional and hybrid heterostructures. These structures' layers are positioned between the sensing medium and the BK7-prism to simulate SPR. The suggested biosensor is represented in the modified Kretschmann configuration. Without using approximations, this method is accurate, simple, and straightforward for estimating the reflectivity of structures made up of several thin film layers^18,27^. In this regard, all layers of the structures examined in this research are arranged along the z-axis, as depicted in Fig. 1. In this case, the developed SPR biosensor is estimated to operate with a P-polarized wave at a wavelength of 633 nm. To attain the highest potential sensitivity for the proposed SPR biosensor, it is imperative to implement the most effective techniques. So, several conventional and hybrid heterostructures are investigated and shown in Table 1. The refractive indices of the various layers used in traditional and hybrid heterostructures are also displayed in Table 2.

To maximize surface plasmon penetration into the sensing medium, and get R_min_ close to zero, which is thought to be required for the development of any SPR sensor, Ag-layer thickness, type, and layer thickness of the dielectric materials are optimized. Furthermore, the comparison between the Ag-bulk metal and Ag-nanocomposite will be achieved to show their effect on the SPR sensor's performance. Additionally, the impact of the Ag-nanocomposite's host materials and nanoparticle volume fraction on the performance parameters is explored.

These whole analyses for the proposed structures have been done using eleven structures, as shown in Table 1. By keeping an eye on changes in the resonance angle of the reflectance dip, which was brought on by variations in the sensing medium's refractive index between 1.33 and 1.34, the properties of these structures were investigated using SPR.

### Optimization of the conventional SPR multilayer sensors

**Figure 3:**
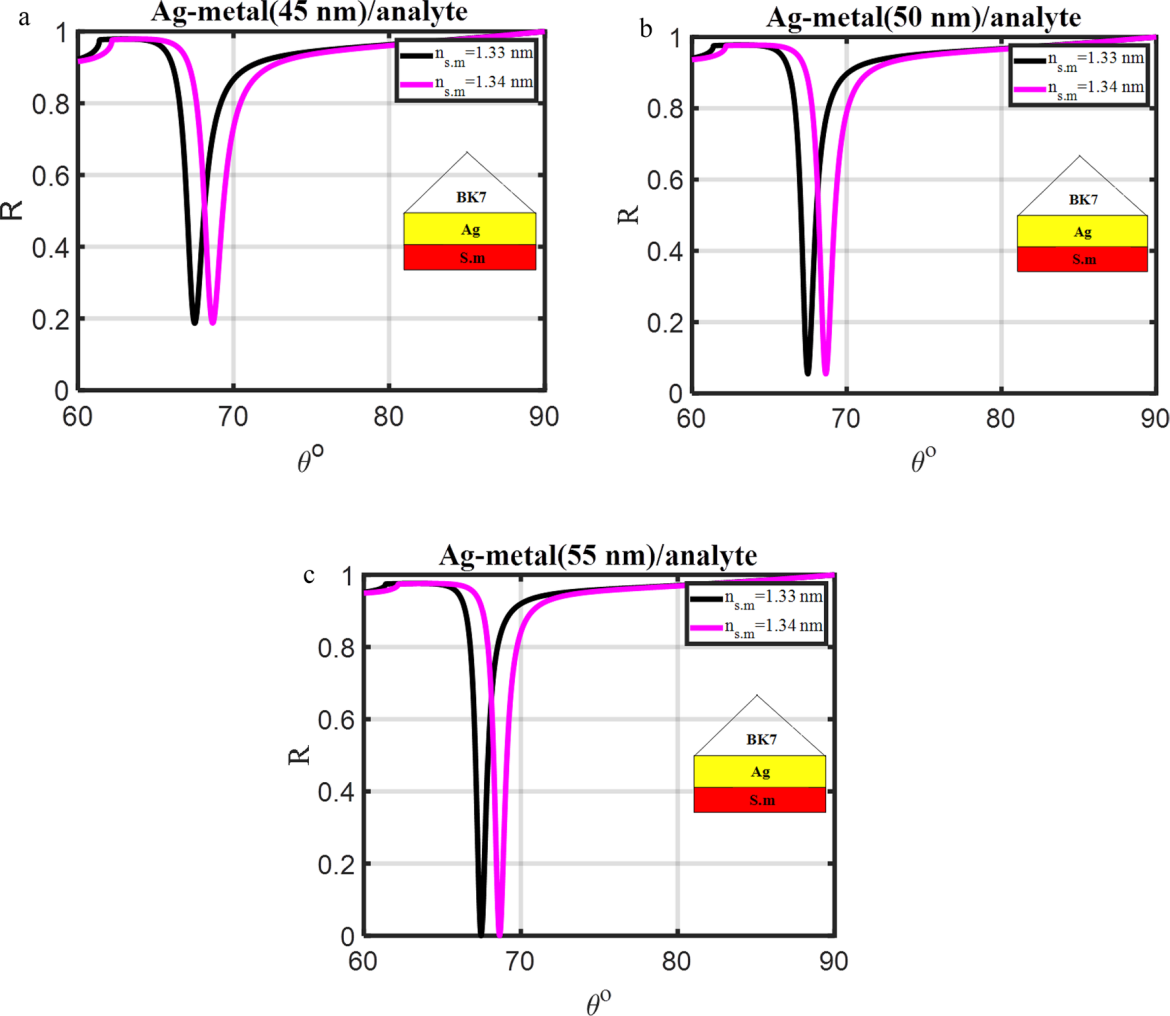
Resonance angle-dependent reflectance of traditional SPR design (prism/Ag/s.m) with a sample of 1.33 and 1.34 refractive indices. Reflectance spectra show two solid line curve colors that, respectively, correspond to the refractive indices of 1.33 and 1.34 of the sensing medium.

**Figure 4:**
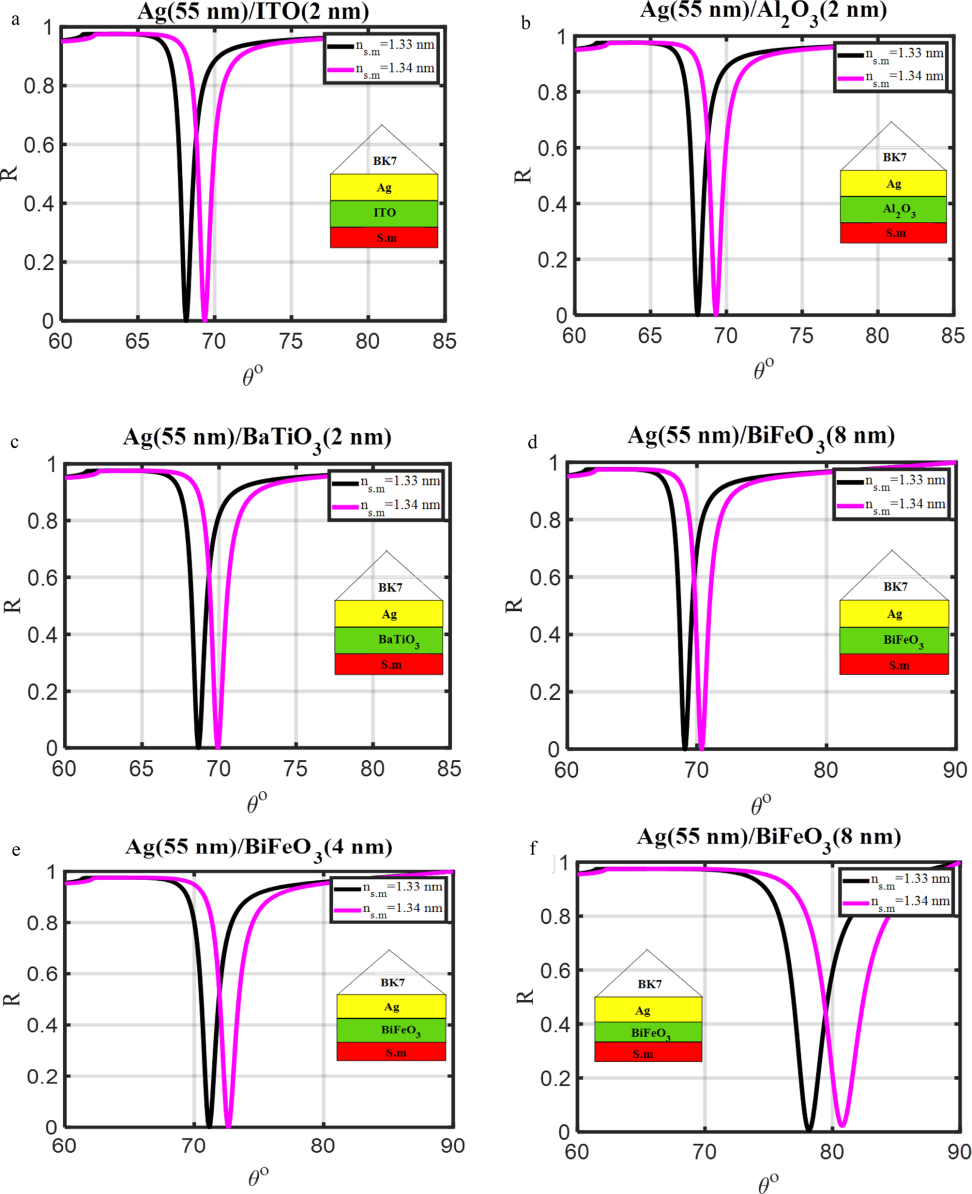
Resonance angle-dependent reflectance of traditional SPR design (prism/Ag/dielectric materials/s.m) with a sample of 1.33 and 1.34 refractive indices. Reflectance spectra show two solid line curve colors that, respectively, correspond to the refractive indices of 1.33 and 1.34 of the sensing media.

**Figure 5:**
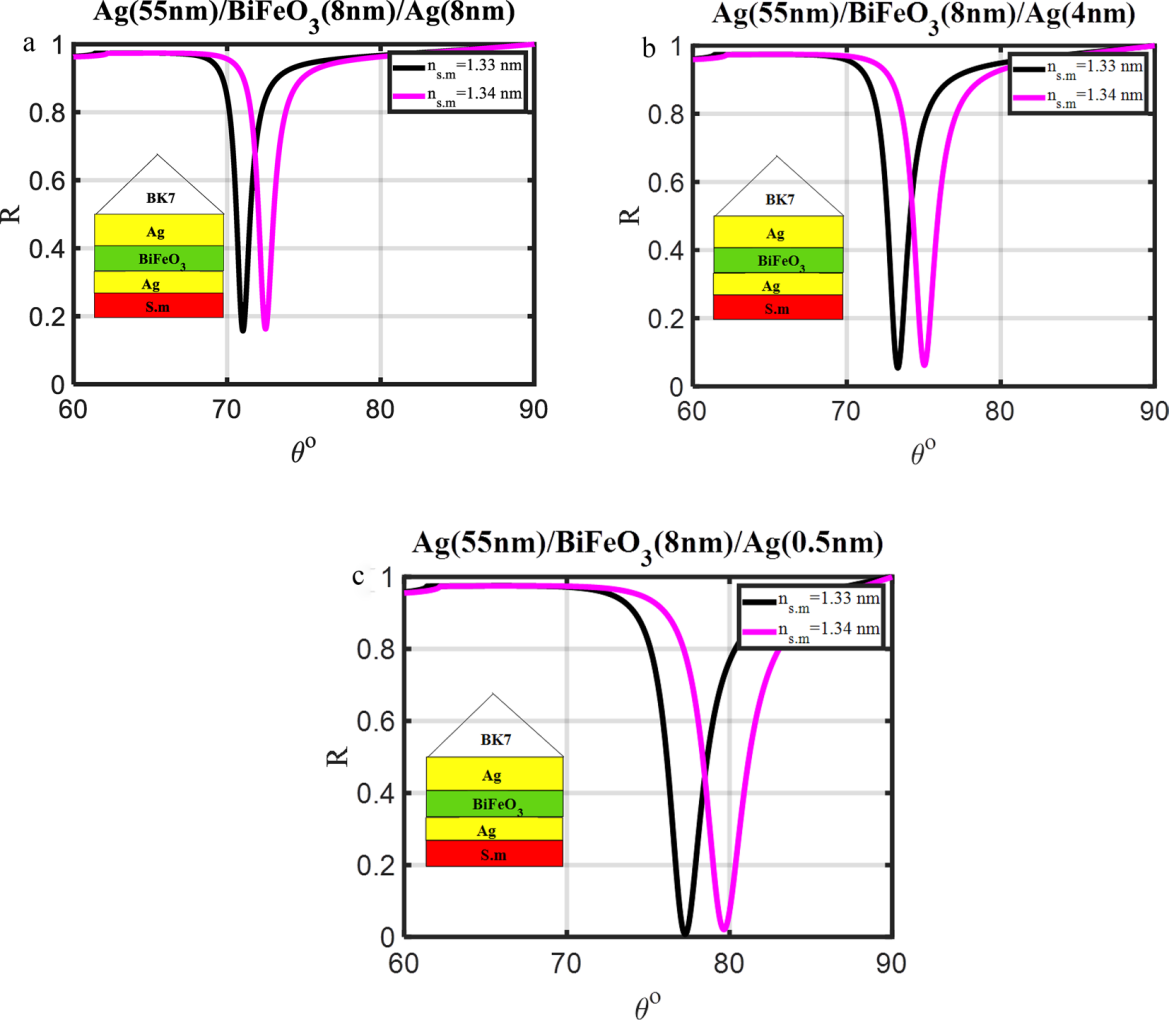
Resonance angle-dependent reflectance of traditional SPR design (prism/Ag/BiFeO_3_/Ag/s.m) with a sample of 1.33 and 1.34 refractive indices. Reflectance spectra show two solid line curve colors that, respectively, correspond to the refractive indices of 1.33 and 1.34 of the sensing media.

The initial step in using SPR setups as a sensing tool is applied to confirm that the prism with the silver coating can exhibit a resonance dip. Angle interrogation was the method used to investigate the resonance dip, and the rotational stage shown in Fig. 2 was utilized to change the angle of incidence. When the light energy was coupled into the plasmons of the metal-dielectric contact, a resonance dip was observed. The angle-dependent reflectivity of the three typical SPR structures (prism/Ag/s.m, prism/Ag/dielectric materials/s.m, and prism/Ag/BiFeO_3_/Ag/s.m) that have been injected with an analyte for refractive index modification (Δn = 0.01) is shown in Figs. 3, 4, and 5.

### Optimization of the first design (prism/Ag/s.m)

The SPR for the structure (prism/Ag/s.m) with a thickness (45 nm) occurs at angles θsp = 67.503° and θsp = 68.659° for ns.m = 1.33 RIU and ns.m = 1.34 RIU, respectively as clear in Fig. 3a. As illustrated in Fig. 3a–c, the silver layer thickness (d_Ag_) is optimized in the initial stages of the investigations to get Rmin close to zero. It was noted that the Rmin and FWHM decreased from (0.186 and 1.410) to (zero, and 0.795) at ns.m = 1.33 RIU. In addition, the Rmin, and FWHM decrease from (0.187, and 1.515) to (0.004, and 0.870) at ns.m = 1.34 RIU, respectively. Moreover, the sensitivity varies from 115.55 to 120.06°/RIU for the d_Ag_ changing from 45 to 55 nm, respectively, as shown in Table 3. In Fig. 3a–c, the resonance angles for the rose's various Ag layer thicknesses are shown concerning the refractive index value of the sensing medium. The resonance angle at which the resonance condition is satisfied can be explained by Eq. (5): for different thick Ag layers, increasing the sensing medium's refractive index also increases the real component of the SP wave's wave vector (KSPW), which in turn increases the resonance angle^3^. However, this modification causes a change in the layer's dielectric constant since it increases the Ag layer's thickness from 45 to 55 nm (in step 5 nm). The Ag layer's thickness also helps to establish the resonance condition at which the resonance angle is occurring. As a result, the Ag layer's thickness that will be utilized in the following calculation will be 55 nm.

**Table 3:** The structural details, R_min_, FWHM, and sensitivity of the conventional multilayer designs, concerning the variation in the refractive index of the sensing medium from 1.33 to 1.34.

Design	Fig	Thickness (nm)	${\text{S}}=\frac{x{\Delta \theta }}{{\Delta n}(0.01)}\left(\circ/\text{RIU}\right)$	n_s.m_ = 1.33		n_s.m_ = 1.34	
				R_min_	FWHM	R_min_	FWHM
Prism/Ag/S.m	3a 3b 3c	d_Ag_ = 45 d_Ag_ = 50 d_Ag_ = 55	115.55 117.06 120.06	0.186 0.054 0	1.410 1.050 0.795	0.187 0.054 0.004	1.515 1.255 0.870
Prism/Ag(55 nm)/dielectric material (2 nm)/S.m	4a 4b 4c 4d	ITO Al_2_O_3_ BaTiO_3_ BiFeO_3_	120.78 120.15 125.47 132.12	0 0 0 0	0.882 0.977 0.955 1.035	0 0 0 0	0.946 0.940 1.029 1.1084
Prism/Ag(55 nm)/BiFeO_3_/S.m	4e 4f	d_Bi_ = 4 d_Bi_ = 8	147.63 259.88	0 0.005	1.347 2.726	0 0.022	1.469 3.165
Prism/Ag(55 nm)/BiFeO_3_(8 nm)/Ag1/S.m	5a 5b 5c	d_Ag1_ = 8 d_Ag1_ = 4 d_Ag1_ = 0.5	148.05 171.91 241.66	0.156 0.053 0.007	1.096 1.570 2.49	0.162 0.061 0.020	1.201 1.752 2.919

## Optimization of the second design (prism/Ag/dielectric materials/s.m)

The performance of the SPR sensor drops as a result of the Ag layer's solidity issue. Additionally, there are issues related to oxidation and corrosion. To ensure optimal performance, it is necessary to utilize a protective layer above the Ag layer^38^. Therefore, different types of dielectric materials (ITO, Al_2_O_3_, BaTiO_3_, and BiFeO_3_) have been tested over the Ag layer to get the best dielectric material that gives the best sensor performance, as shown in Fig. 4a–f. These structures (2–5) in Table 1 belong to the waveguide (WG) structure which is constructed by adding dielectric layers to either side of a metallic layer^4^. The thickness of the all-dielectric layers (the four different cases) was set to 2 nm, and Table 2 displays each layer's refractive index. The SPR propagation real part constant of the wave vector changes whenever a dielectric layer with a thickness of 2 nm comes into contact with an Ag layer. This raises the resonance angle of the proposed SPR sensor. Table 3 shows the sensitivity of the SPR sensor with the fixed Ag layer's thickness and the used dielectric layer. It was noted that; SPR sensor sensitivity is greater with any dielectric layer present than when no dielectric material is present. This is due to the large resonance angle shift, which raises the sensitivity of the SPR sensor according to Eq. (14). It is clear from Fig. 4a–f and Table 3, that the sensitivity increases from 120.06°/RIU without using a dielectric layer to 132.12°/RIU with using a dielectric layer. After comparing different dielectric materials, BiFeO_3_ is the best dielectric material that gives high sensitivity. BiFeO_3_ layer thickness optimization is accomplished by carefully controlling the greatest amount of surface plasmon penetration into the sensing medium. This is a crucial element in the design of an SPR biosensor. The thickness of BiFeO_3_ material changes from 2 to 9 nm as shown in Fig. 4d–f. The resonant angle shift grows together with the rise in BiFeO_3_ thickness. Where, at the ideal thickness of the dielectric layer, Strong SPRs might result from constructive light interferences that maximize energy transfer^39^. Additionally, the thickness of this layer is chosen to increase the SPR sensor's performance. Table 3 shows that the SPR sensor's sensitivity rises from 132.12°/RIU to 159.88°/RIU. Therefore, the BiFeO_3_ layer's thickness utilized in the proposed structure should be 8 nm.

### Optimization of the third design (prism/Ag/BiFeO_3_/Ag/s.m)

The proposed structure is now given a new Ag layer, and it changes into structure-6, which is made of metal-dielectric-metal (MDM), as illustrated in Table 1. By linking (SPR) and (WG) modes, this substructure is used to produce long-range surface plasmons (LRSPs) with lower absorption losses^12,39,40^. Greater electric field strengths are produced by the LRSPP while absorption losses are lower. The appropriate thickness of the metal layer in the sensor should be acquired since the Ag layer thickness is one of the most important parameters that impact the SPR biosensor's performance. High sensitivity and minimal reflection (R_min_) are desired characteristics for the SPR biosensor for improved performance. According to Maurya and Prajapati^34^, the thickness of the Ag layer that corresponds to the smallest reflectivity is the ideal thickness. The sensitivity at the minimum reflectivity has a value that is nearly identical to the highest sensitivity. As shown in Fig. 5a–c, with a decrease in the Ag layer's thickness from 8 to 0.5 nm, the R_min_ decreased from (0.156) to (0.007) at n_s.m_ = 1.33 RIU. Also, the R_min_ decreases from (0.162) to (0.020) at n_s.m_ = 1.34 RIU, respectively. In addition, the sensitivity increases from 148.05 to 241.66°/RIU, respectively, as shown in Table 3. Therefore, in the proposed construction, an Ag layer with a thickness of 0.5 nm is chosen for optimal results.

### Optimization of the hybrid heterostructure angular SPR sensor

The metal (silver) material has a higher sensitivity for SPR sensing, as was already indicated, but due to its weak chemical stability, it cannot be developed further. The biosensor's sensitivity has recently been enhanced using several methods, such as transforming the silver layer into silver nanoparticles embedded in the host dielectric materials^21,35,36^.

**Figure 6:**
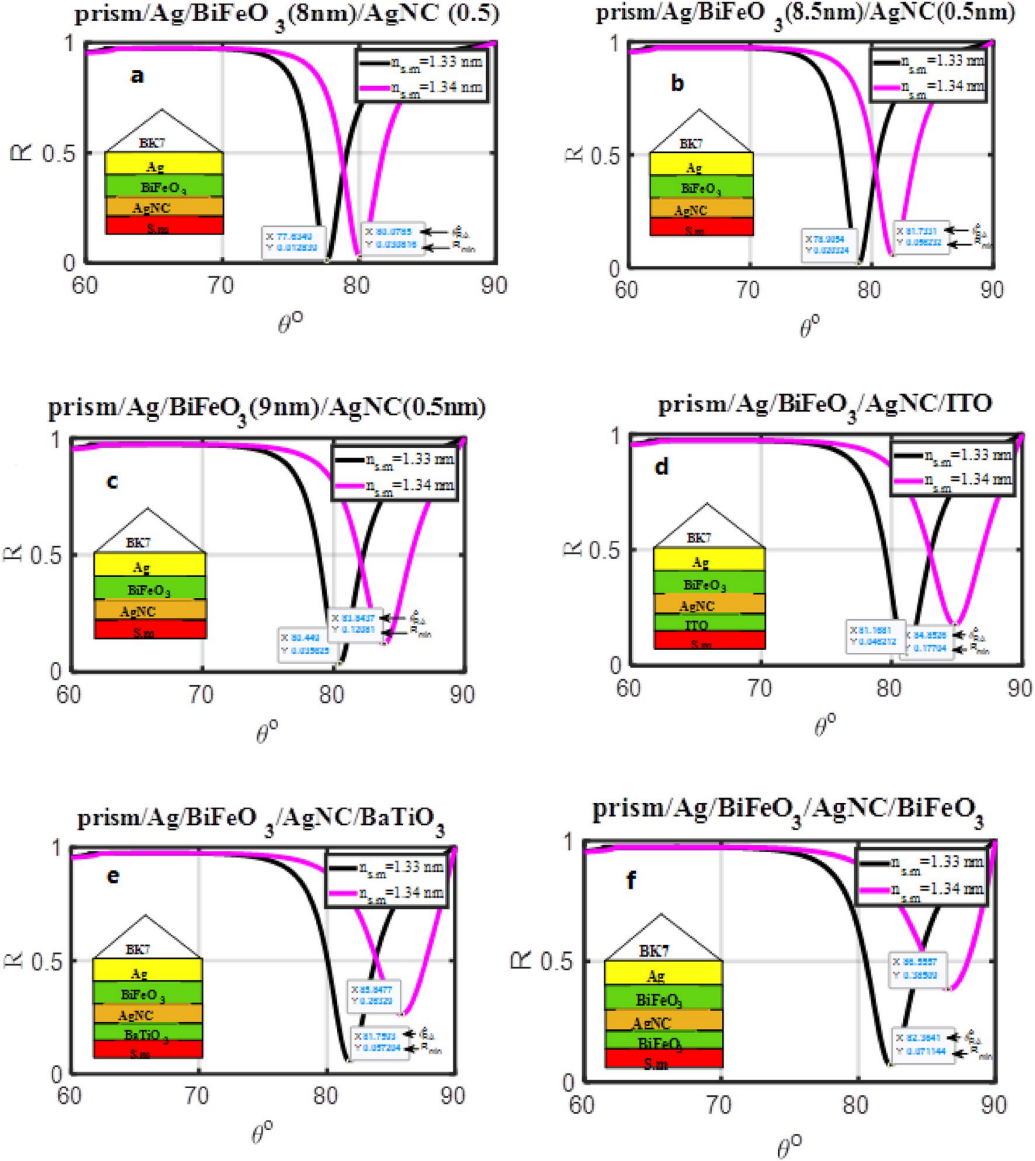
Resonance angle-dependent reflectance of hybrid SPR heterostructures with a sample of 1.33 and 1.34 refractive indices. Reflectance spectra show two solid line curve colors that, respectively, correspond to the refractive indices of 1.33 and 1.34 of the sensing medium.

The silver nanocomposite layer was used in place of the silver Layer (No. 3) to further increase sensitivity. The hybrid heterostructure used as an angular SPR sensor (prism/Ag (55 nm)/BiFeO_3_ (8 nm)/Ag nanocomposite (0.5 nm)/s.m.) is shown to have angle-dependent reflectivity in Fig. 6a. This structure allows for the coupling of LRSP and DWG modes. These modes contribute to the development of strong evanescent field strengths and deeper penetration. It was observed that the sensitivity increased from 241.66°/RIU to 249.31°/RIU. Figure 6a–c shows the effect of the BiFeO_3_ layer's thickness on the proposed hybrid heterostructure's sensitivity. It was observed that with an increase in the thickness from 8 nm to 9 nm, the sensitivity increases from 249.31°/RIU to 348.03°/RIU, as shown in Table 4. This demonstrates that one of the criteria in the construction of an SPR sensor composed of a hybrid heterostructure is the optimization of the thickness of the dielectric material. The biosensing capacities of biosensors formed of hybrid multilayer structures can be further improved by adding a dielectric thin film to the metal nanocomposite surface to develop guided-wave surface plasmon resonance (GWSPR) based biosensors.

**Table 4:** The structural details, R_min_, FWHM, and sensitivity of the SPR sensor constructed of a hybrid heterostructure, concerning the variation in the refractive index of the sensing medium from 1.33 to 1.34.

Design	Fig	Thickness (nm)	${\text{S}}=\frac{{\Delta \theta }}{{\Delta n}(0.01)}\left(\circ/\text{RIU}\right)$	n_s.m_ = 1.33		n_s.m_ = 1.34	
				R_min_	FWHM	R_min_	FWHM
Prism/Ag(55 nm)/BiFeO_3_/AgNC(0.5 nm)/S.m	4a 4b 4c	d_Bi_ = 8 d_Bi_ = 8.5 d_Bi_ = 9	249.31 287.87 348.02	0.012 0.020 0.035	2.730 3.069 3.481	0.030 0.056 0.120	3.123 3.669 4.438
Prism/Ag(55 nm)/BiFeO_3_(9 nm)/AgNC(0.5 nm)/dielectric material (0.5 nm)/S.m	4d 4e 4f	ITO BaTiO_3_ BiFeO_3_	378.45 408.45 419.16	0.045 0.057 0.071	3.722 3.929 4.141	0.177 0.263 0.385	4.901 5.283 5.414

After that, a layer of dielectric material was put between the silver nanocomposite layer and the structure's sensing medium (prism/Ag (55 nm)/BiFeO_3_ (9 nm)/Ag nanocomposite (0.5 nm)/dielectric material/s.m). Because inserting any dielectric material has enhanced the SPR and offered further corrosion resistance, this construction improved the angular shift in the reflectance dip as well as the sensitivity. Fig. 6d–f shows the comparison between three dielectric materials (ITO, BaTiO_3_, and BiFeO_3_) with a thickness of 0.5 nm. It was noted that; the BiFeO_3_ material achieved the highest sensitivity (419.16°/RIU) compared to other materials.

All of the aforementioned substructures are characterized by having very few values for both the R_min_ and FWHM compared to all published research, and the difference between the values is very small^4,7^. Therefore, the sensitivity was the important performance parameter for optimizing our SPR sensor.

### Optimization of the host material and volume fraction of nanoparticles used in silver nanocomposite

Equation (3) states that silver nanoparticles embedded in a host dielectric substance are what give the nanocomposite layer its refractive index. Therefore, the refractive index of both silver nanoparticles and host material is the cornerstone of the refractive index of the nanocomposite layer. Therefore, the refractive index of the host material is added to the parameters that determine the resonance condition. In addition, the surface-to-volume ratio of the reinforcing component (volume fraction of nanoparticles) in nanocomposites makes them different from conventional composite materials^37^. Where the increasing of the volume fraction ensures the complete transfer of incident light to surface plasmon in the prism which results in a smaller R_min_^38^. So, the type of host material and volume fraction of the nanoparticle must be optimized for our angular SPR hybrid heterostructure (Prism/Ag(55 nm)/BiFeO_3_(9 nm)/AgNC(0.5 nm)/BiFeO_3_ (0.5 nm)/s.m).

**Figure 7:**
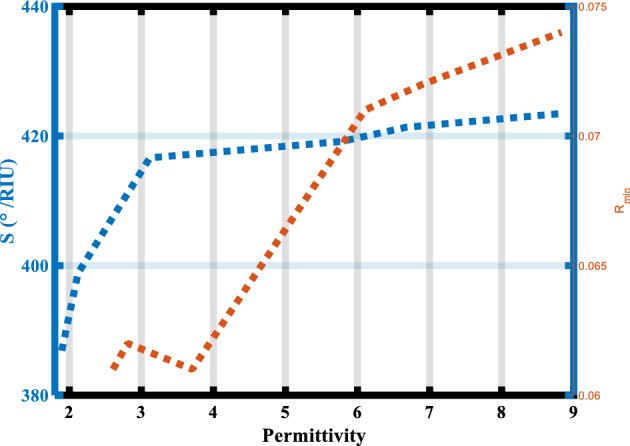
The sensitivity and the R_min_ versus the permittivity of the host dielectric material.

The impact of the host dielectric material type on the proposed SPR sensor's sensitivity will be examined in the subsequent phase. Where the BaTiO_3_ material is used on all aforementioned substructures as a host material. Table 5 shows different types of host dielectric materials (MgF_2_, SiO_2_, AL_2_O_3_, BaTiO_3_, TiO_2_, and BiFeO_3_) arranged according to their value of permittivity (refractive index)^2^. Figure 7 shows the impact of the permittivity of host dielectric materials on the sensitivity and the R_min_ of our sensor. It is noted from this Figure, that with increases in the value of permittivity from 1.896 to 8.809, the sensitivity increases from 386.88°/RIU to 423.45°/RIU, and the value of R_min_ is almost constant and changed by a very small value (0.001). Concluded from this study, BiFeO_3_ is the best host material used as a host material in our SPR hybrid heterostructure.

**Table 5:** The permittivity of the host dielectric material used at wavelength 633 nm.

Materials	Permittivity (ε_d_)	${\text{S}}=\frac{{\Delta \theta }}{{\Delta n}(0.01)}\left(\circ/\text{RIU}\right)$	R_min_ (n_s.m_ = 1.33)	Ref
MgF_2_	1.896	386.88	0.061	^39^
SiO_2_	2.122	398.86	0.062	^40^
AL_2_O_3_	3.118	416.64	0.061	^41^
BaTiO_3_	5.780	419.16	0.071	^30^
TiO_2_	6.674	421.35	0.072	^42^
BiFeO_3_	8.809	423.45	0.073	^43^

**Figure 8:**
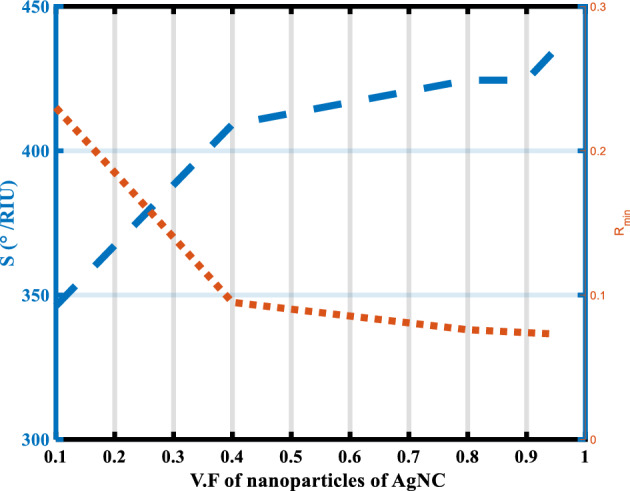
The sensitivity and the R_min_ versus the volume fraction of the nanoparticles in nanocomposite material.

The effect of the volume fraction of nanoparticles on the R_min_ and the sensitivity of our SPR hybrid heterostructure is shown in Fig. 8 and Table 6. Where the used value of the volume fraction of nanoparticles in all the above structures equals (0.9). It is noted from Fig. 8 and Table 6, with the increase in the volume fraction from (0.1 to 0.95), the sensitivity increases from 346.23°/RIU to 435.29°/RIU, and the value of R_min_ decreases from 0.230 to 0.073. This behavior agrees with experimental research. According to Mandal et al.^21^, it can be seen that the plasmon-derived resonance of these crystals becomes stronger and wider as the volume percentage of silver Nano crystallites increases. As silver Nano crystal loading in the SiO_2_ matrix increases, it should be observed that the resonance peak shifts to longer wavelengths. This observation is consistent with TEM studies, which show that when the loading of silver crystallites rises, the size of the nanocrystallites grows and their size distribution widens^21^. Therefore, the optimum value of the volume fraction of nanoparticles is 0.95. Finally, the optimized of our angular SPR hybrid heterostructure is considered as (Prism/Ag(55nm)/BiFeO_3_(9nm)/AgNC(0.5nm)/BiFeO_3_ (0.5 nm)/S.m) with optimization parameters of the nanocomposite layer (BiFeO_3_ as a host material and the volume fraction of nanoparticles is 0.95), and the performance parameters are display in Table 7.

**Table 6:** The different values for the volume fraction of nanoparticles in nanocomposite materials.

Volume fraction of nanoparticle	${\text{S}}=\frac{{\Delta \theta }}{{\Delta n}(0.01)}\left(\circ/{RIU}\right)$	R_min_ (n_s.m_ = 1.33)
1 × 10^–1^	346.23	0.230
4 × 10^–1^	409.16	0.095
8 × 10^–1^	424.47	0.076
9 × 10^–1^	424.47	0.074
9.5 × 10^–1^	435.29	0.073

**Table 7:** The performance parameters of optimized our angular SPR sensor.

Optimized our hybrid heterostructure	n_s.m_ (RIU)	${\uptheta }_{{\text{Res}}}{^\circ }$	R_min_	FWHM (degree)	${\text{S}}\left(\circ/\text{RIU}\right)$	${\text{QF}}$	${\text{FoM}}$ ${{\text{RIU}}}^{-1}$	${\text{D}}.{\text{L}}$ × 10^–4^ ${\text{RIU}}$	SNR	${\text{S}}.{\text{R}}$ (degree)
Prism/Ag(55 nm)/BiFeO_3_(9 nm)/AgNC(0.5 nm)/BiFeO_3_(0.5 nm)/S.m	1.33	82.531	0.073	4.121	–	20.026	–	–	–	–
	1.34	86.884	0.423	5.29	435.29	16.424	82.285	6.076	0.822	0.264

### Detection of different polluted water samples:

**Figure 9:**
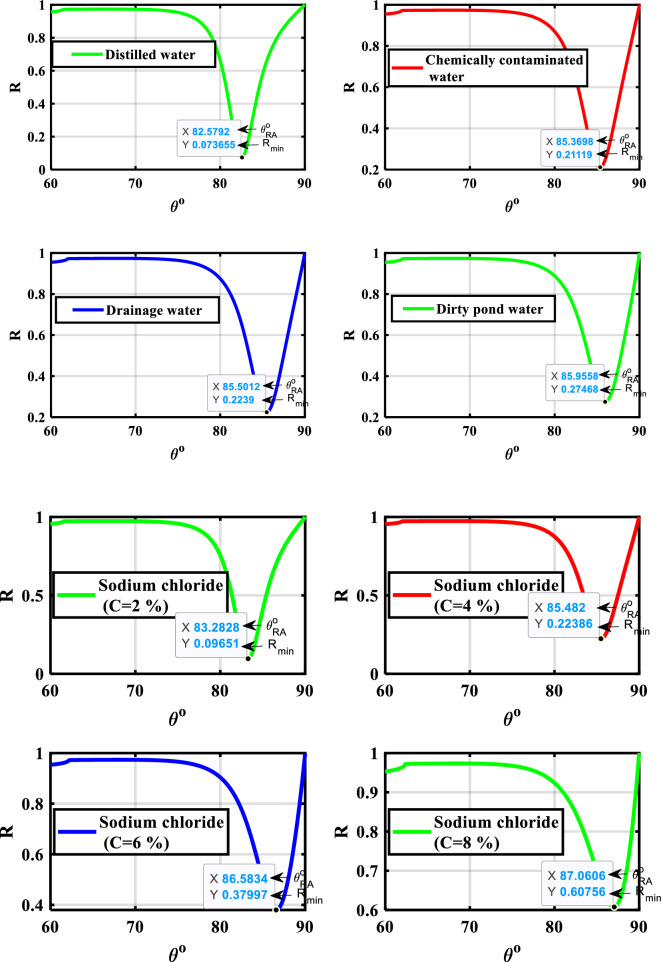
Resonance angle-dependent reflectance of hybrid SPR heterostructures with a different contaminated water, and different concentration of the sodium chloride.

Our angular SPR hybrid heterostructure sensor's direct link with the sensing medium makes it suitable for usage as a monitor and detection of different contaminated water. The different contaminated water samples, such as chemically contaminated water, drainage water, and dirty pond water (polluted with mud and animal excrement), as well as other samples made in the lab with varying weights, include sodium chloride in weights of (2, 4, 6, and 8)% mg in 10 ml of water. An Abbe refractometer was used in the lab to measure the refractive index of these contaminated samples as shown in Table 8^44^. The suggested angular SPR hybrid heterostructure sensor is shown in Fig. 9 to be capable of identifying several samples of contaminated water (the sensing medium) with RI values ranging from 1.330 to 1.34. This Figure indicates that increasing the sensing medium's refractive index results in a redshift, which corresponds to the resonance angle shifting to longer angles. This phenomenon can be attributed to the resonance condition of SPWs. Specifically, when the sensing medium's refractive index is sufficiently high, the real component of the propagation constant of the SPWs also increases, which allows for resonance at longer angles, according to Eq. (1). It noted that the resonance angle for the distilled water, chemically contaminated water, drainage water, and Dirty Pond water are 82.579°, 85.369°, 85.501°, and 85.955°. For the different concentrations of sodium chloride (0, 2, 4, 6, and 8), the resonance angles were found to be 82.579°_,_ 83.282°, 85.482°, 86.583°, and 87.060°, respectively.

**Table 8:** The refractive index and concentration of the different samples of contaminated water were measured by an Abbe refractometer device^44^.

_Samples_	_RI of sensing medium (ns.m)_	_Concentration (%)_
_Distilled water_	_1.33_	**–**
_Chemically contaminated water_	_1.3367_	**–**
_Drainage water_	_1.337_	**–**
_Dirty pond water_	_1.338_	**–**
_Sodium chloride_	_1.332_	_2_
_Sodium chloride_	_1.337_	_4_
_Sodium chloride_	_1.3395_	_6_
_Sodium chloride_	_1.34_	_8_

**Figure 10:**
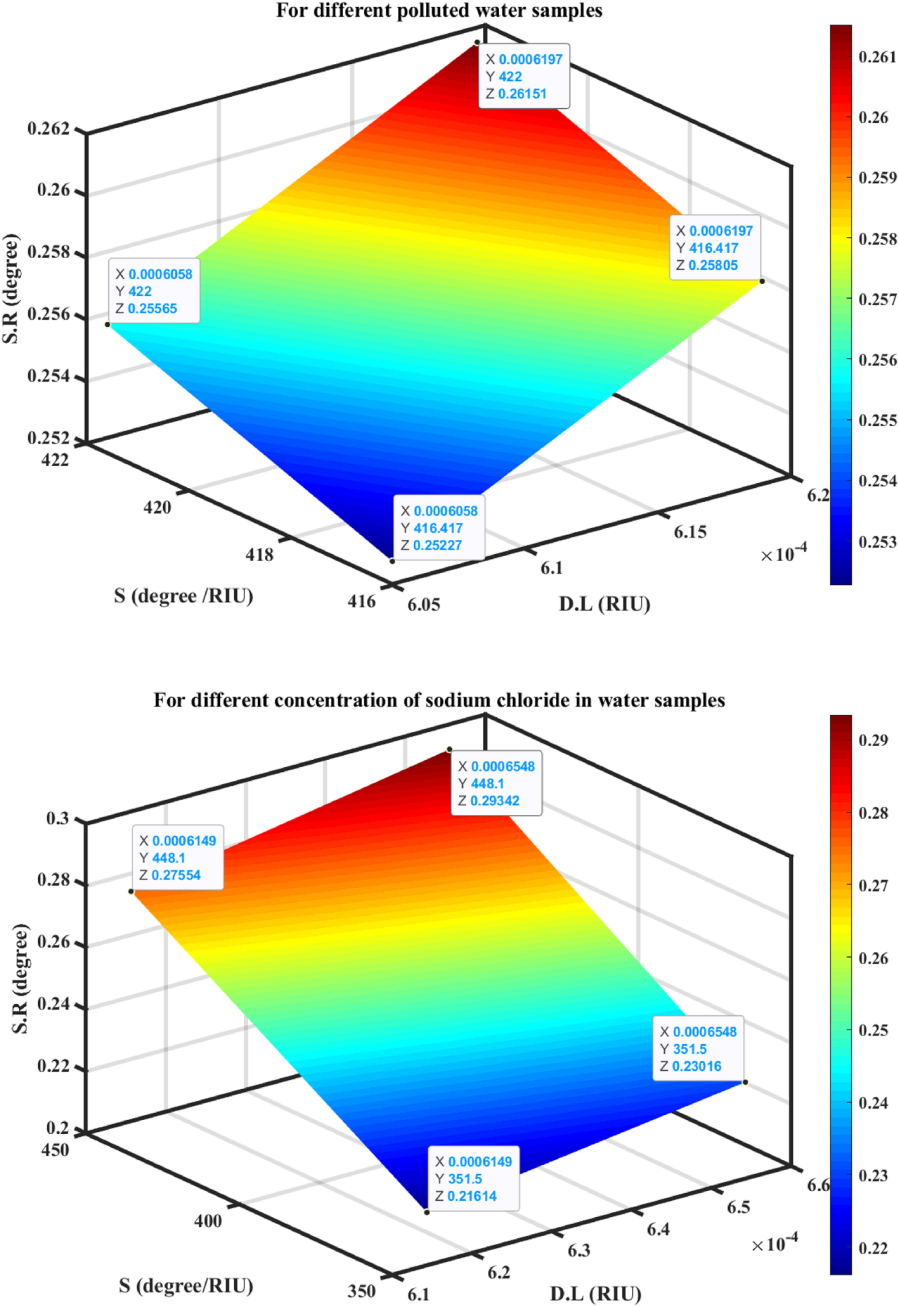
The detection limit, sensitivity, and sensor resolution of the angular SPR sensor.

The suggested SPR sensor's performance parameter values for detecting various levels of pollution in water samples are shown in Table 8 and Fig. 10. Equations (15–19) are used to calculate, consequently, the sensitivity, quality factor, figure of merit, detection limit, and sensor resolution. To measure the sensitivity for the detection of different polluted water samples, the difference between the resonance angles of the polluted water and the distillate (pure) water was calculated. As previously indicated, great sensitivity is achieved by achieving a significant shift in the resonance angle (during in-phase interrogation) with only a minor change in the sample's concentration and refractive index^3^. It was found that, the resonance angle shift ($\Delta {\uptheta }_{{\text{RA}}}$) increases from 2.79° to 3.376° for different polluted water samples and increases from 0.703° to 4.481° for different concentrations of sodium chloride in water samples. The resulting high sensitivity of 422°/RIU for polluted water samples, and 448.1°/RIU for the concentration of sodium chloride in water samples. The other performance parameters are listed in Table 9. Furthermore, Fig. 10 illustrates how the sensitivity impacts other factors like the Detection Limit and Sensor Resolution. Equation (19) states that as sensitivity rises, sensor resolution rises and detection limit falls. For various types of polluted water or various sodium chloride concentrations, this behavior is illustrated in Table 9 in detail.

**Table 9:** Performance parameters of the optimized hybrid heterostructure (prism/Ag/BiFeO_3_/Ag-nanocomposite/BiFeO_3_/Ag-nanocomposite/BiFeO_3_/sensing medium) loaded with different polluted water samples.

n_s.m_ (RIU)	∆n_s.m_ (RIU)	${\uptheta }_{{\text{Res}}}{^\circ }$	R_min_	FWHM (degree)	${\Delta\uptheta }_{{\text{Res}}}^{^\circ }$	$\mathrm{S }\left(\circ/\text{RIU}\right)$	${\text{QF}}$	${\text{FoM}}$ ${{\text{RIU}}}^{-1}$	${\text{D}}.{\text{L}}$ × 10^–4^ ${\text{RIU}}$	SNR	${\text{S}}.{\text{R}}$ (degree)
1.33	–	82.579	0.073	4.139	–	–	19.951	–	–	–	–
1.3367	6.7 × 10^–3^	85.369	0.211	5.046	2.79	416.417	16.918	82.524	6.058	0.552	0.252
1.337	7 × 10^–3^	85.501	0.223	5.059	2.922	417.428	16.900	82.511	6.06	0.577	0.252
1.338	8 × 10^–3^	85.955	0.274	5.230	3.376	422	16.434	80.688	6.197	0.645	0.261
1.332 (2%)	2 × 10^–3^	83.282	0.096	4.323	0.703	351.5	19.264	81.309	6.149	0.162	0.216
1.337 (4%)	7 × 10^–3^	85.482	0.223	5.230	2.903	414.714	16.344	79.295	6.305	0.555	0.261
1.3395 (6%)	9.5 × 10^–3^	86.583	0.379	5.396	4.004	421.473	16.045	78.108	6.401	0.742	0.269
1.34 (8%)	10 × 10^–3^	87.060	0.607	5.689	4.481	448.1	15.303	78.766	6.548	0.787	0.284

### Comparison with previous work

We next compare our findings in Table 10 with many related studies in this field to show the improved performance and sensitivity of our sensor compared to the previously published sensors. This table demonstrated that the suggested sensor generates an enhanced sensitivity of 448.1$\circ/\text{RIU}$, which is noticeably greater than most of the listed works in Table 10.

**Table 10:** Comparison of the sensitivity of our angular SPR hybrid heterostructure sensor with the previous SPR sensors.

Ref	Configuration of SPR	Based on	Operating wavelength (nm)	Sensitivity
^35^	Ni/ZnO nanocomposite assisted with graphene	Kretschmann configuration	633 (angular interrogation)	378.34$\circ/\text{RIU}$
^45^	Hetero-structured air/MoS2/nanocomposite/MoS2/graphene	Otto configuration	633 (angular interrogation)	200$\circ/\text{RIU}$
^46^	Prism (BK7), *silver* (Ag), titanium dioxide (TiO_2_), hybrid inorganic–organic halide *perovskites* (MAPbBr_3_) and graphene (Gr) layers	Kretschmann configuration	632.8 (Angular interrogation)	224$\circ/\text{RIU}$
^47^	A nanocomposite-based fiber optics sensor with platinum nanoparticles	Kretschmann configuration	Depend on (wavelength interrogation)	Not present
^48^	BK7 prism/Ag/BiFeO_3_/graphene/analyte	Kretschmann configuration	633	293$\circ/\text{RIU}$
^49^	Ag metallic layer coated with chitosan–graphene oxide nanocomposite	By using SPR setup	850 nm	1.38◦ ppm^ − ^^1^
^50^	titanium, silver, graphene, photonic crystal, and a sensing layer	Not present	633	72$\circ/\text{RIU}$
Proposed work	Prism/Ag/BiFeO_3_/AgNC/BiFeO_3_/S.m	Kretschmann configuration	633	448$\circ/\text{RIU}$

## Conclusion

The usage of the Kretschmann configuration based on the angular interrogation is suggested for this design in this study, which explains how hybrid heterostructure angular SPR can be applied as an upgraded sensor. A nanocomposite layer made of silver and BiFeO_3_ used as a plasmonic material has been identified as a potential method to improve the performance of the suggested sensor. The proposed technique is analyzed based on the transfer matrix method to quantitatively compute the reflectance spectra of the proposed hybrid heterostructure angular SPR sensor at operating wavelength 633 nm. The examination of the suggested sensor's performance is conducted regarding the following parameters, sensitivity, quality factor, figure of merit, detection limit, and sensor resolution. To attain the utmost level of sensitivity, optimal values for various parameters are identified, taking into account the impact of the metallic layer's thickness, type, and the dielectric layer's thickness. Based on the numerical findings, it was determined that the suggested angular SPR sensor demonstrated a sensitivity up to the value of 448.1$\circ/\text{RIU}$ at the optimal state. The sensor that was produced demonstrated a significant increase in sensitivity, as it exhibited a 54% improvement when compared with the conventional SPR biosensors. Finally, the outcomes evinced that the proposed hybrid heterostructure angular SPR sensor outperforms the biosensors expounded in the preceding literature, rendering it well-suited for deployment in the monitoring and detection of diverse contaminated water.

## Data Availability

Requests for materials should be addressed to Arafa H. Aly.
